# A phase 2 study of mobocertinib as first-line treatment in Japanese patients with non-small cell lung cancer harboring *EGFR* exon 20 insertion mutations

**DOI:** 10.1007/s10147-024-02588-y

**Published:** 2024-08-27

**Authors:** Kiyotaka Yoh, Koichi Azuma, Hidetoshi Hayashi, Makoto Nishio, Kenichi Chikamori, Eiki Ichihara, Yasutaka Watanabe, Takayuki Asato, Tadayuki Kitagawa, Robert J. Fram, Yuichiro Ohe

**Affiliations:** 1 Department of Thoracic Oncology, National Cancer Center Hospital East, 6-5-1 Kashiwanoha, Kashiwa-Shi, Chiba, 277-8577 Japan; 2 Division of Respirology, Neurology, and Rheumatology, Department of Internal Medicine, Kurume University School of Medicine, 67, Asahi-Machi, Kurume, Fukuoka 830-0011 Japan; 3 Faculty of Medicine, Department of Medical Oncology, Kindai University, 377-2 Ōnohigashi,, Ōsakasayama-Shi, Ōsaka-Fu 589-0014 Japan; 4 Department of Thoracic Medical Oncology, The Cancer Institute Hospital of Japanese Foundation for Cancer Research, 3-8-31, Ariake, Koto, Tokyo 135-8550 Japan; 5 Department of Medical Oncology, National Hospital Organization Yamaguchi-Ube Medical Center, 685 Higashi Kiwa, Ube, Yamaguchi 755-0241 Japan; 6 Center for Clinical Oncology, Okayama University Hospital, 2-5-1 Shikata-Cho, Kita-Ku, Okayama, 700-8558 Japan; 7 Department of Thoracic Oncology, Saitama Cancer Center, 780 Komuro, Inamachi, Kitaadachi-Gun, Saitama, 362-0806 Japan; 8 Oncology Clinical Research Department, Oncology Therapeutic Area Unit for Japan and Asia, Takeda Pharmaceutical Company Limited, 1-1, Doshomachi 4-Chome, Chuo-Ku, Osaka, 540-8645 Japan; 9 Biostatistics, Japan Development Center, Takeda Pharmaceutical Company Limited, 1-1, Doshomachi 4-Chome, Chuo-Ku, Osaka, 540-8645 Japan; 10 Takeda Development Center Americas, Inc, 40 Landsdowne Street, Cambridge, MA 02139 USA; 11 Department of Thoracic Oncology, National Cancer Center Hospital, 5-1-1 Tsukiji, Chuo-Ku, Tokyo 104-0045 Japan

**Keywords:** *EGFR* exon 20 insertion mutations, Mobocertinib, NSCLC, Tyrosine kinase inhibitor

## Abstract

**Background:**

Mobocertinib is a novel, synthetic, orally administered tyrosine kinase inhibitor that inhibits many activated forms of epidermal growth factor receptor (EGFR), including those containing exon 20 insertion (ex20ins) mutations. This study aimed to assess the efficacy of mobocertinib in Japanese patients with locally advanced or metastatic non-small cell lung cancer (NSCLC) harboring *EGFR* ex20ins mutations.

**Methods:**

This was a phase 2, open-label study. Patients with NSCLC harboring *EGFR* ex20ins mutations who had not had previous systemic treatment received mobocertinib 160 mg once daily. The primary endpoint was the confirmed objective response rate. A planned interim analysis was completed for the first 14 patients with a centrally confirmed *EGFR* ex20ins mutation, with enrollment stopped if the number of patients with an objective response was five or fewer.

**Results:**

In total, 33 patients were enrolled into the study (63.6% women; median age: 66 years). At the interim analysis, the objective response rate evaluated by a central independent review committee was 28.6% (4/14, 90% confidence interval: 10.4–54.0); therefore, enrollment was stopped for futility. In the full analysis set, the objective response rate was 18.2% (6/33, 95% confidence interval: 7.0–35.5); of the six responders, one patient (3.0%) had a complete response and five patients (15.2%) had partial responses. The most common treatment-related adverse events were diarrhea, paronychia, stomatitis, and nausea.

**Conclusion:**

Although study enrollment was terminated early owing to futility, our results showed modest activity of mobocertinib in Japanese patients with NSCLC with *EGFR* ex20ins mutations with no additional safety concerns.

**Supplementary Information:**

The online version contains supplementary material available at 10.1007/s10147-024-02588-y.

## Introduction

Specific genetic lesions that drive the proliferation of cancer cells, such as those resulting in activation of certain tyrosine kinases, render many cancers highly sensitive to therapeutic agents that inhibit the affected kinase. These include activating mutations in the epidermal growth factor receptor gene (*EGFR*) which, according to a 2016 systematic review and meta-analysis, are found in around one-third of patients with non-small cell lung cancer (NSCLC) globally [1]. However, prevalence does vary geographically, with high mutation rates reported in Asian populations [1–3]. There are multiple classes of activating *EGFR* mutations, which vary widely in their degree of sensitivity to available tyrosine kinase inhibitors (TKIs). Given that inhibition of wild-type EGFR in normal tissues is associated with dose-limiting toxicities, substantial clinical benefit in NSCLC has been associated with TKIs that inhibit specific, activated variants of EGFR more potently than they inhibit wild-type EGFR [4].

The most common activating mutations in *EGFR* are in-frame deletions in exon 19 and a L858R substitution in exon 21, which together account for about 90% of all *EGFR* activating mutations [5–7]. The remaining 10–15% of de novo *EGFR* mutations comprise a cluster of in-frame insertions in exon 20 found in approximately 2% of all cases of NSCLC [1, 5, 8], as well as other rarer *EGFR* ‘uncommon’ point mutations [6]. Patients with NSCLC with *EGFR* exon 20 insertions exhibit similar clinical characteristics (e.g., age, smoking status, lung cancer subtype) to patients carrying common *EGFR* mutations [5, 9]. However, unlike mutations in exons 19 or 21, almost all *EGFR* exon 20 insertions confer in vitro and primary clinical resistance to the TKIs erlotinib, gefitinib, and afatinib [10–12]. Patients with exon 20 insertions are therefore more likely to benefit from novel targeted TKI therapies that selectively inhibit these particular *EGFR* mutations [4].

Although five TKIs targeting *EGFR* mutations (afatinib, dacomitinib, erlotinib, gefitinib, and osimertinib) are approved in Japan, none are recommended in the Japan Lung Cancer Society clinical practice guidelines for the treatment of patients with *EGFR* exon 20 insertion mutations [13]. Mobocertinib is a novel, synthetic, orally administered TKI developed to address the limitations of these existing therapies. In clinical studies, mobocertinib has been shown to strongly inhibit many activated forms of EGFR, including those containing exon 20 activating insertions, uncommon activating mutations, or common activating mutations (exon 19 deletions and L858R) with or without the T790M resistance mutation [14]. A US phase 1/2 study of mobocertinib in platinum-pretreated patients with NSCLC with *EGFR* exon 20 insertion mutations determined that the maximum tolerated dose was 160 mg once daily (QD) [15]. A subsequent phase 1 study in Japan confirmed that this dose was also tolerable in a Japanese patient population [16]. Results from the Japanese study indicated that mobocertinib had a manageable safety profile in patients with NSCLC and provided pharmacokinetic data to support 160 mg QD as the recommended dose for phase 2 clinical studies in Japanese patients.

Here, we report the results of a phase 2 study designed to evaluate the efficacy of mobocertinib 160 mg QD in Japanese patients with locally advanced or metastatic NSCLC whose tumors harbor *EGFR* exon 20 insertion mutations and who have not previously received systemic treatment for locally advanced or metastatic disease.

## Patients and methods

### Study design

This was a phase 2, open-label, uncontrolled trial conducted at 17 sites in Japan with patients recruited between February 4, 2019 and March 10, 2021 (ClinicalTrials.gov: NCT03807778). The trial design included a 2–3-week screening period, a treatment period with a planned estimated average of 10–12 cycles (each cycle lasting 28 days), and a follow-up period. Mobocertinib 160 mg QD was self-administered until patients experienced progressive disease (PD) requiring an alternate therapy (in the opinion of the investigator), intolerable toxicity, or another discontinuation criterion.

The study protocol and associated documentation were reviewed by institutional review boards at each site. The study was carried out in compliance with the Declaration of Helsinki, the International Council for Harmonisation Good Clinical Practice guidelines, and all applicable local regulations. All patients provided written informed consent before enrollment.

### Patients

Eligible patients were 20 years of age or older with histologically or cytologically confirmed locally advanced, recurrent, or metastatic (stage IV) NSCLC, measurable disease by RECIST v1.1, an Eastern Cooperative Oncology Group (ECOG) performance status (PS) of 0 to 1, a minimum life expectancy of at least 3 months, and adequate renal, hepatic, and bone marrow function. Patients could not have received prior systemic treatment for locally advanced or metastatic disease and had to have a locally documented *EGFR* in-frame exon 20 insertion mutation (either alone or in combination with other *EGFR* mutations [excluding exon 19 deletions or L858R] or human epidermal growth factor receptor 2 mutations). All patients had to have adequate tumor tissue available for central laboratory confirmation of *EGFR* exon 20 insertion mutation, which was confirmed using the Oncomine Dx Target Test. Key exclusion criteria were diagnosis of a primary malignancy other than NSCLC; spinal cord compression or leptomeningeal disease; significant, uncontrolled, or active cardiovascular disease; radiotherapy in the 14 days before the first dose of mobocertinib; or known active brain metastases. Patients were also excluded if they had received a moderate or strong cytochrome P450 3A inhibitor or inducer in the 10 days before the first dose of mobocertinib.

### Study assessments

Disease assessment at screening included imaging of the chest, abdomen, pelvis, and brain using appropriate radiologic procedures (computed tomography scans or magnetic resonance imaging with contrast). Disease assessment was then performed at 8-week intervals (on Day 28 [± 7 days] of every even-numbered cycle) through cycle 14, and every three cycles thereafter until PD. Partial response (PR) and complete response (CR) were confirmed by a repeat tumor imaging assessment at least 4 weeks after the date the response was first documented.

Health-related quality of life (HRQoL) was assessed using the European Organisation for Research and Treatment of Cancer (EORTC) Quality of Life Questionnaires, including lung cancer-specific module 13 (QLQ-LC13) v.3.0 [17]. Raw scores were converted into scale scores ranging from 0 to 100; for the functional scales and the global health status scale, higher scores represent better HRQoL, whereas for the symptom scales lower scores represent better HRQoL.

Treatment-emergent adverse events (hereafter referred to as AEs unless otherwise specified) were collected from study drug initiation to 30 days after study drug discontinuation or before the initiation of new anticancer therapy (whichever came first) and coded according to Medical Dictionary for Regulatory Activities (MedDRA) version 23.0. Severity grades were defined by the Common Terminology Criteria for Adverse Events version 5.0.

### Study endpoints

The primary endpoint was the confirmed objective response rate (ORR; the proportion of patients who were confirmed to have achieved CR or PR), as assessed by the independent review committee (IRC) per RECIST v1.1. Confirmed responses were responses that persisted on repeat imaging for at least 4 weeks after initial response. Secondary endpoints included: confirmed ORR, as assessed by the investigator; duration of response (DOR), as assessed by the IRC and the investigator; disease control rate (DCR; the percentage of patients with best response of CR, PR, or stable disease [SD] of 42 days or longer) as assessed by the IRC and the investigator per RESIST v1.1; progression-free survival (PFS) as assessed by the IRC and the investigator; overall survival (OS); and patient-reported outcomes including the EORTC QLQ-LC13. Patients who discontinued study treatment in the absence of PD continued to have post-treatment PFS and OS follow-up assessments. Safety endpoints included AEs, laboratory values, vital signs, and physical examination findings.

### Statistical methods

A sample size of 26 patients with centrally confirmed *EGFR* exon 20 insertion mutations was chosen based on an expected true ORR of 60% in the treatment-naive population and an ORR threshold response rate of 35%. This would give over 80% power to rule out an uninteresting rate of 35% in this population, with a 1-sided α of 0.05. The expected true ORR was based on the results of a preliminary efficacy analysis of mobocertinib, which demonstrated an ORR of approximately 60% in patients with unresectable advanced or recurrent NSCLC harboring *EGFR* exon 20 insertion mutations who had received prior chemotherapy. Also taking into consideration the general health status of chemotherapy-naïve patients, the expected response rate was therefore set at 60%. In chemotherapy-naïve patients with unresectable advanced or recurrent NSCLC positive for *EGFR* mutations, first-line treatment with platinum-based chemotherapy has been reported to have response rates ranging from 14.9 to 47.3% [18–25]; therefore the threshold ORR was set at 35% for our study.

The full analysis set (FAS) and safety population both included all patients who received at least one dose of mobocertinib. The centrally confirmed population (CCP) was defined as the first 26 patients with confirmed *EGFR* exon 20 insertion mutation by central test who received at least one dose of mobocertinib. An interim analysis for both futility and efficacy was planned based on the primary endpoint after the first 14 centrally confirmed patients had the opportunity to complete the ‘cycle 7, Day 1’ disease assessment. If the number of patients with a confirmed objective response was five or fewer, enrollment would be stopped for futility. If the number of patients with a confirmed objective response was nine or more, mobocertinib would be considered efficacious in this population.

For the primary analysis, a point estimate and 90% 2-sided exact confidence interval (CI) were calculated for the IRC-assessed confirmed ORR. For secondary analysis of the primary endpoint, the IRC-assessed confirmed ORR and 95% 2-sided exact CI were calculated using the CCP and the FAS. Secondary endpoints were also assessed in both the CCP and the FAS. For time-to-event endpoints, the Kaplan–Meier method was used; PFS, OS, and DOR were computed at 12 and 24 months.

## Results

### Patients

**Fig. 1:**
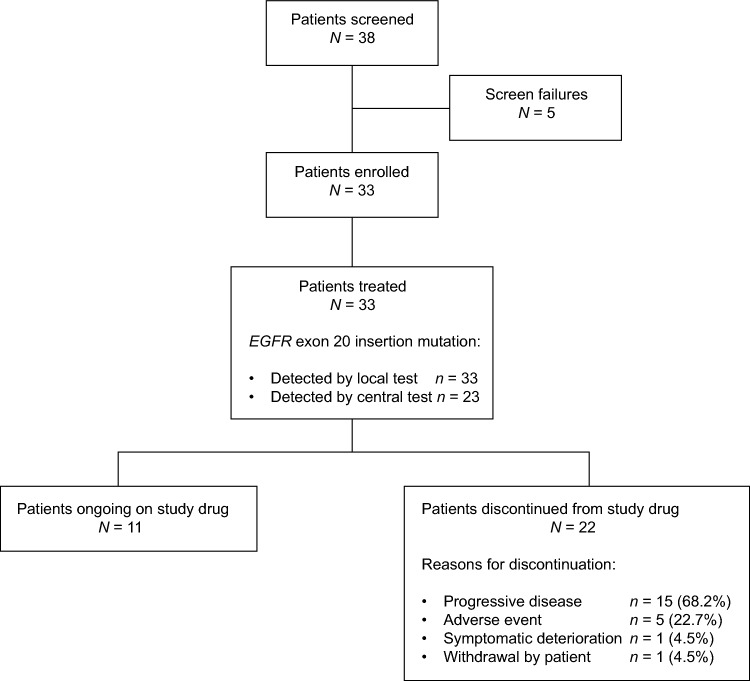
Patient disposition. *EGFR* epidermal growth factor receptor

In total, 38 patients were screened and 33 enrolled into the study (Fig. 1). The median age was 66 years, 63.6% (21/33) of patients were women, 48.5% (16/33) had stage IVA disease at screening, and 63.6% (21/33) of patients had an ECOG PS of 0 (Table 1). There were 23 (69.7%) patients who had their *EGFR* exon 20 insertion mutation confirmed by central test; those whose mutation could not be confirmed centrally had either defective tumor tissue samples or insufficient quantities of DNA for testing.

**Table 1:** Patient baseline characteristics (safety population)

	Mobocertinib 160 mg QD (*N* = 33)
Age, years	
Median	66.0
Minimum, maximum	39, 84
Age category, years, *n* (%)	
< 50	4 (12.1)
50– < 65	12 (36.4)
65– < 75	11 (33.3)
≥ 75	6 (18.2)
Sex, *n* (%)	
Male	12 (36.4)
Female	21 (63.6)
NSCLC stage at screening, *n* (%)	
IVA	16 (48.5)
IVB	15 (45.5)
Other^a^	2 (6.1)
Time since initial diagnosis, months	
Median	1.90
Minimum, maximum	0.5, 166.0
ECOG PS, *n* (%)	
0	21 (63.6)
1	12 (36.4)
CNS involvement at screening, *n* (%)	
Yes	16 (48.5)
No	17 (51.5)
Histopathological classification of NSCLC, *n* (%)	
Adenocarcinoma	33 (100.0)
Smoking history, *n* (%)	
Never	17 (51.5)
Former	16 (48.5)
*EGFR* exon 20 insertion mutation	
Detected by local test, *n* (%)	33 (100.0)
Confirmed by central test, *n* (%)	23 (69.7)

^a^Includes postoperative recurrence and postoperative relapse

*CNS* central nervous system, *ECOG* Eastern Cooperative Oncology Group, *EGFR* epidermal growth factor receptor gene, *NSCLC* non-small cell lung cancer, *PS* performance status, *QD* once daily

All 33 patients received at least one dose of study drug. Here, data are presented as of November 8, 2021, at which point 11 patients were still receiving treatment. Overall, the median (range) duration of study drug exposure was 8.3 (0.4–18.0) months. The median (range) dose intensity was 114.8 (52–160) mg/day and the median (range) relative dose intensity was 71.7% (33–100). In total, eight patients (24.2%) experienced significant protocol deviations, which were most frequently related to concomitant medications (4 patients [12.1%]). None of the protocol deviations were considered to have had an impact on the efficacy or safety conclusions of the study.

### Interim analysis

At the interim analysis (data cut-off: February 24, 2021), the IRC-assessed confirmed ORR for the first 14 patients in the CCP (primary endpoint) was 28.6% (4/14, 90% CI 10.4–54.0), and all four responders had PR as the best overall response. Because the number of patients with a confirmed objective response was five or fewer, enrollment was stopped for futility and the interim analysis became the primary analysis. Enrollment had not been suspended during the evaluation of the first 14 patients in the CCP, resulting in 33 patients being included in the FAS.

### Efficacy outcomes

**Fig. 2:**
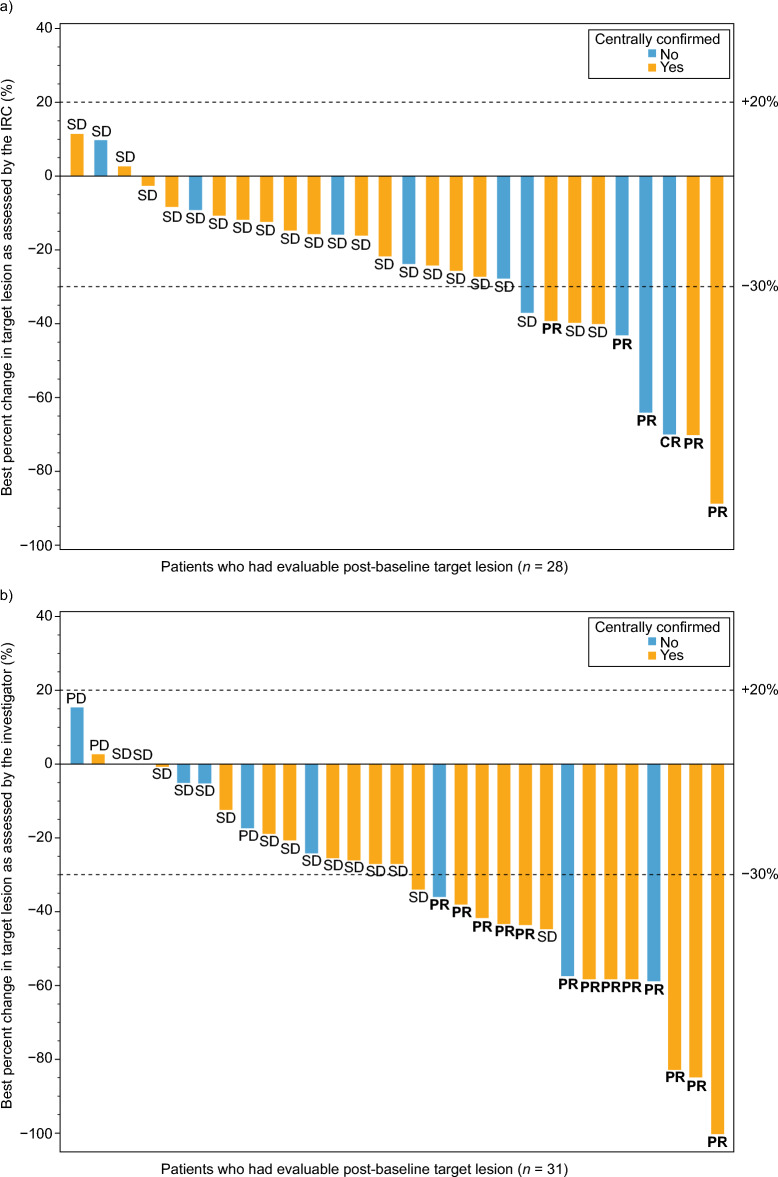
Best percent change in target lesion as assessed by **a** the IRC^a^ and **b** the investigator^b^ (FAS). ^a^Of the 33 patients in FAS, five patients were excluded from the waterfall plot (three patients had no measurable target lesions by IRC assessment; two patients had no proper imaging assessment during study treatment). ^b^Of the 33 patients in FAS, two patients were excluded from the waterfall plot (had no proper imaging assessment during study treatment). *CR* complete response, *FAS* full analysis set, *IRC* independent review committee, *PD* progressive disease, *PR* partial response, *SD* stable disease

A summary of key final efficacy results as assessed by the IRC and the investigator for the FAS and the CCP is shown in Table 2. The IRC-assessed confirmed ORR in the FAS was 18.2% (6/33, 95% CI 7.0–35.5); of the six responders, one patient (3.0%) had CR and five patients (15.2%) had PR as best overall response (Table 3). The IRC-assessed confirmed ORR in the CCP was 13.0% (3/23, 95% CI 2.8–33.6); all three responders had PR as best overall response. One patient who was evaluated to have PR at the interim analysis had their response changed to SD at secondary analysis following a change in IRC reviewer. The investigator-assessed confirmed ORR in the FAS was 39.4% (13/33, 95% CI 22.9–57.9) and all responders had PR as best overall response (Table 3). In the CCP, this was 43.5% (10/23, 95% CI 23.2–65.5) and all 10 responders had PR as best overall response. Figure 2 shows the best percent change in target lesion as assessed by the IRC (panel a) and the investigator (panel b) for both the FAS and the CCP.

**Table 2:** Summary of key efficacy results

Endpoint	Assessor	FAS (*N* = 33)		CCP^a^ (*N* = 23)	
		Responders, *n/N*	Point estimate (95% CI)	Responders, *n/N*	Point estimate (95% CI)
ORR	IRC	6/33	18.2% (7.0–35.5)	3/23	13.0% (2.8–33.6)
	Investigator	13/33	39.4% (22.9–57.9)	10/23	43.5% (23.2–65.5)
DOR^b^	IRC	–	7.4 months (3.7–NE)	–	7.4 months (7.3–NE)
	Investigator	–	7.4 months (3.6–NE)	–	6.6 months (1.8–NE)
DCR	IRC	28/33	84.8% (68.1–94.9)	19/23	82.6% (61.2–95.0)
	Investigator	28/33	84.8% (68.1–94.9)	21/23	91.3% (72.0–98.9)
PFS^b^	IRC	–	9.2 months (6.7–NE)	–	9.3 months (7.4–NE)
	Investigator	–	7.4 months (4.6–NE)	–	9.2 months (6.2–NE)

^a^Patients whose tumor specimen had been retrospectively confirmed to have an *EGFR* exon 20 insertion mutation by an analytically validated central test

^b^Data show median values

*CCP* centrally confirmed population, *CI* confidence interval, *DCR* disease control rate, *DOR* duration of response, *FAS* full analysis set, *IRC* independent review committee, *NE* not evaluable, *ORR* objective response rate, *PFS* progression-free survival

**Table 3:** Confirmed ORR (IRC- and investigator-assessed) (FAS)

Measure	Mobocertinib 160 mg QD (*N* = 33)	
	IRC	Investigator
Confirmed ORR (CR + PR), *n* (%)	6 (18.2)	13 (39.4)
95% CI^a^	7.0–35.5	22.9–57.9
Best overall response, *n* (%)		
CR	1 (3.0)	0 (0.0)
PR	5 (15.2)	13 (39.4)
SD	22 (66.7)	15 (45.5)
Non-CR/non-PD	3 (9.1)	0 (0.0)
PD	0 (0.0)	4 (12.1)
NE	2 (6.1)	1 (3.0)

^a^Data show the exact Clopper–Pearson CI of the percentage

*CI* confidence interval, *CR* complete response, *FAS* full analysis set, *IRC* independent review committee, *NE* not evaluable, *ORR* objective response rate, *PD* progressive disease, *PR* partial response, *QD* once daily, *SD* stable disease

The median DOR in the FAS was 7.4 months (95% CI 3.7–not evaluable [NE]) as assessed by the IRC and 7.4 months (95% CI 3.6–NE) as assessed by the investigator (Table 2). The DCR in the FAS was 84.8% (95% CI 68.1–94.9) as assessed by the IRC and 84.8% (95% CI 68.1–94.9) as assessed by the investigator (Table 2).

**Fig. 3:**
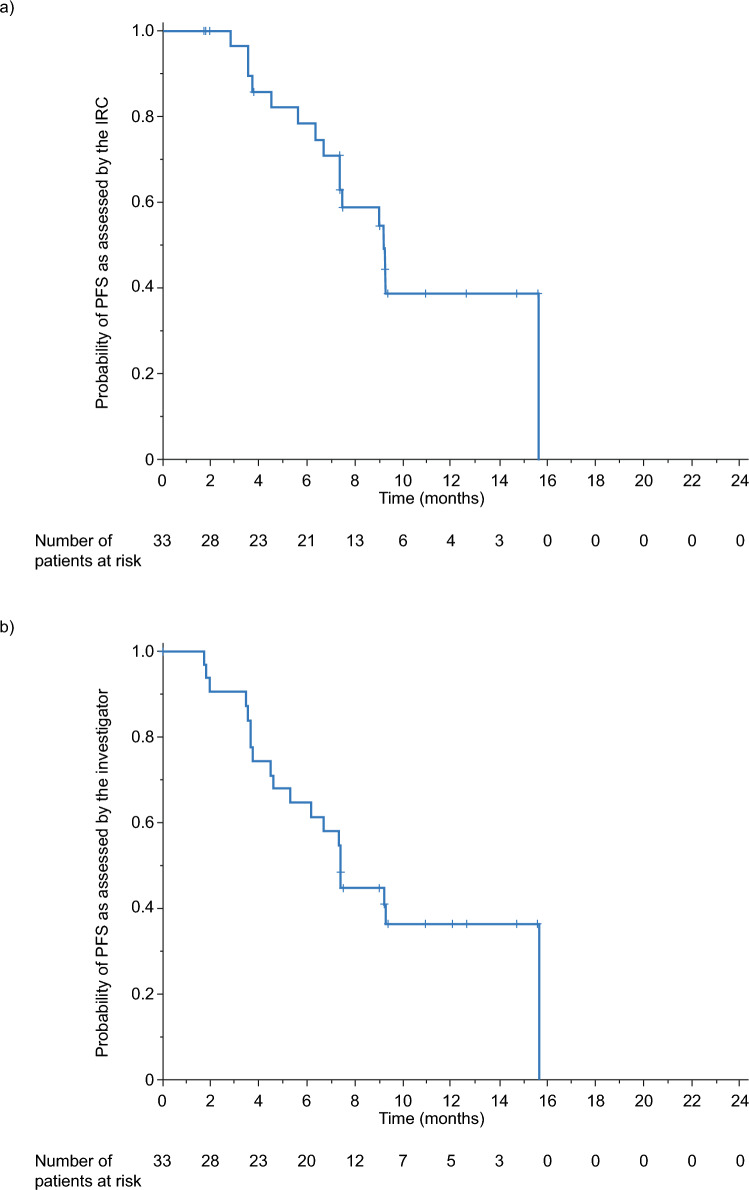
PFS as assessed by **a** the IRC and **b** the investigator (FAS). *FAS* full analysis set, *IRC* independent review committee, *PFS* progression-free survival

**Fig. 4:**
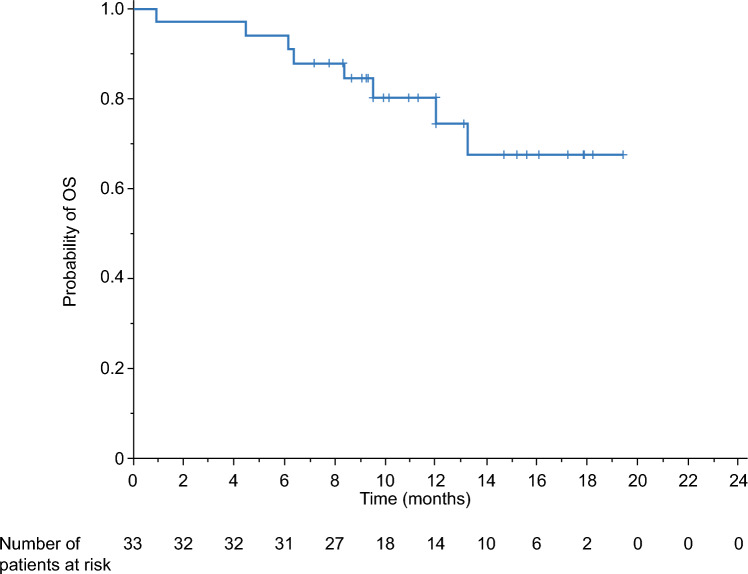
Overall survival (FAS). *FAS* full analysis set, *OS* overall survival

The median PFS in the FAS was 9.2 months (95% CI 6.7–NE) as assessed by the IRC and 7.4 months (95% CI 4.6–NE) as assessed by the investigator (Table 2). Kaplan–Meier estimates of PFS rates in the FAS at 6 and 12 months were 78.3% and 38.8%, respectively, as assessed by the IRC, and 64.5% and 36.3%, respectively, as assessed by the investigator (Fig. 3). Kaplan–Meier plots of OS in the FAS are presented in Fig. 4. The Kaplan–Meier estimated OS rates at 6 and 12 months were 93.9% and 80.3%, respectively. In total, eight patients (24.2%) died during the study and the median OS follow-up (12.1 months) was not reached.

**Fig. 5:**
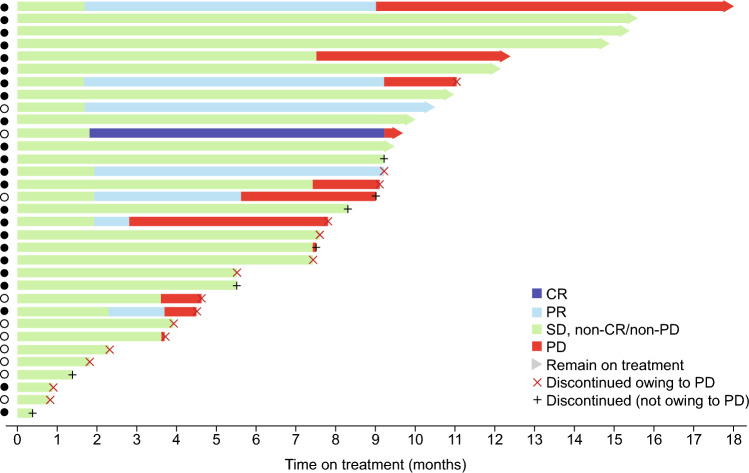
Swimlane plot of IRC-assessed response profile by patient (FAS). Patients included in the CCP are shown by a filled circle; patients not included in the CCP are shown by an unfilled circle. *CCP* centrally confirmed population, *CR* complete response, *FAS* full analysis set, *IRC* independent review committee, *PD* progressive disease, *PR* partial response, *SD* stable disease

Swimlane plots of the IRC-assessed response profiles by patient in the FAS are shown in Fig. 5. In the FAS, 11 patients were still on study treatment at data cut-off, for whom the IRC-assessed best overall responses were CR (1 patient), PR (2 patients), and SD (8 patients).

For the EORTC QLQ-LC13, there was no change from baseline in scores for dyspnea, coughing, hemoptysis, dysphagia, peripheral neuropathy, alopecia, pain in chest, pain in arm or shoulder, and pain (other parts) throughout the study. The mean (standard deviation) subscale score in sore mouth at baseline was 5.0 (12.0), which increased to 35.4 (30.6) at cycle 2. By cycle 6, the mean (standard deviation) score had improved to 17.2 (19.4), and this improvement was maintained throughout the remainder of the study.

### Safety outcomess

An overview of AEs is provided in Supplementary Table S1. All patients had at least one AE and at least one treatment-related AE. The most common AEs were gastrointestinal disorders and skin and subcutaneous tissue disorders (Supplementary Table S2). AEs of grade 3 or higher were observed in 21 (63.6%) patients; the most frequently reported AE of grade 3 or higher was diarrhea (*n* = 7). The most common treatment-related AEs were diarrhea (*n* = 33), paronychia (*n* = 21), stomatitis (*n* = 20), and nausea (*n* = 19).

Overall, 81.8% (27/33) of patients had an AE leading to dose modification, 60.6% (20/33) had an AE leading to dose reduction, and 15.2% (5/33) discontinued treatment owing to an AE. Serious AEs (SAEs) were reported for 11 patients (33.3%). The most common SAE was decreased appetite (*n* = 3); all other SAEs were each reported in one patient. AEs resulting in death were reported in two patients (6.1%; cardiac failure and NSCLC); neither death was considered to be related to the study drug.

## Discussion

This phase 2 study was designed to evaluate the efficacy of mobocertinib 160 mg QD in Japanese patients with locally advanced or metastatic NSCLC whose tumors harbor *EGFR* exon 20 insertion mutations and who had not previously received systemic treatment for locally advanced or metastatic disease. Results from the interim analysis showed that the IRC-assessed confirmed ORR in the first 14 centrally confirmed patients was 28.6% (4/14), which met the predefined criteria for futility and enrollment was terminated early.

Treating patients with NSCLC harboring *EGFR* exon 20 insertion mutations has been challenging owing to the limited efficacy of approved TKIs targeting *EGFR* mutations. The first- and second-generation EGFR TKIs only achieved response rates of approximately 10%, with an estimated median PFS of 1–3 months [26]. According to results from LC-SCRUM-Asia, a large-scale clinico-genomic database, Japanese patients with NSCLC harboring *EGFR* exon 20 insertion mutations treated with classical TKIs in the second-to-fourth line settings after platinum-based chemotherapy reported an ORR of 8% (95% CI 1.7–2.2) with a median PFS of 2.2 months (95% CI 1.6–3.7) [27]. In a phase 1/2 study that evaluated the third-generation EGFR TKI osimertinib (80 mg/day), none of the 12 enrolled patients with NSCLC harboring *EGFR* exon 20 insertion mutations experienced an objective response [28]. Seven (58.3%) and five (41.7%) patients had stable disease and disease progression, respectively, and the median PFS was 3.8 months.

To address these unmet medical needs, mobocertinib was developed to specifically target *EGFR* exon 20 insertion mutations. The antitumor activity of mobocertinib was demonstrated in an international phase 1/2 study that enrolled platinum-pretreated patients with NSCLC harboring *EGFR* exon 20 insertion mutations; the ORR was 28% (95% CI 20–37) and the IRC-assessed median PFS was 7.3 months (95% CI 5.5–9.2) [29]. Based on these results, mobocertinib was granted accelerated approval in the USA in September 2021 [30]. Subsequently, an international, phase 3, open-label study (EXCLAIM-2) compared first-line mobocertinib treatment to platinum-based chemotherapy in patients with locally advanced/metastatic NSCLC positive for *EGFR* exon 20 insertion mutations [31]. That study was terminated early owing to futility following the results of an interim analysis, in which the blinded IRC-assessed median PFS was similar for mobocertinib and platinum-based chemotherapy (9.6 months for both treatment arms).

The selection of ORR as the primary endpoint for our study was based on its utility as a marker of clinical improvement and discussions with Japanese regulatory authorities. Our results are comparable to those from the international mobocertinib studies [29, 31], though caution is warranted when comparing different studies. In our study, the IRC-assessed confirmed ORR was 18.2% (95% CI 7.0–35.5) and the median PFS was 9.2 months (95% CI 6.7–NE). Of note, our investigator-assessed confirmed ORR was 39.4%. The cause of this discrepancy was not clear. However, inconsistencies between assessments of ORR by investigators at local sites and assessments from blinded independent central review in uncontrolled oncology trials are not uncommon and have been reported elsewhere [32, 33].

Considering the available body of evidence, mobocertinib has demonstrated clinical activity in NSCLC harboring *EGFR* exon 20 insertion mutations, where efficacy has been historically low with other EGFR TKIs, albeit in different treatment line settings. The targeted efficacy of mobocertinib is also supported by data from a pre-clinical study, in which mobocertinib more potently inhibited the viability of multiple *EGFR* exon 20 insertion-driven cell lines than other TKIs and demonstrated in vivo antitumor efficacy in patient-derived xenograft animal models [14].

Regarding the tolerability profile of mobocertinib, high rates of dose reduction (60.6%) and dose interruption (81.8%) were observed in our study. However, the reported AEs were consistent with known AEs associated with other EGFR TKIs, and no new safety signals were observed [34]. Diarrhea is commonly observed with other EGFR TKIs, including afatinib, dacomitinib, and osimertinib [35–37], and this was the case with our study as well. The incidence of grade 3 or higher treatment-related diarrhea was 21.2% in our study which was similar to a previous phase 1/2 study [29].

Mobocertinib targets diverse *EGFR* exon 20 insertion mutations with selectivity over *EGFR* wild-type mutations [14]. However, skin-related AEs caused by off-targeting of *EGFR* wild-type mutations cannot be completely avoided. The types and severity of skin-related AEs observed with mobocertinib are consistent with those reported with other EGFR TKIs, with a low frequency of severe events [38].

Limitations of our study include the small sample size and that enrollment was terminated early owing to futility. In addition, relatively high rates of dose reduction and dose interruption, and a reduced relative dose intensity may have influenced the low ORR observed in our study. The study could have benefited from more experienced management of AEs to reduce the impact of these dose modifications. In the EXCLAIM-2 study, the frequency of AEs leading to dose interruption and dose reduction of mobocertinib was lower than in our study, likely reflecting the prophylactic management of AEs [31]. Finally, as *EGFR* exon 20 insertion mutations are highly heterogeneous, with more than 100 known variants [39], it is possible that some subtypes or specific variants may have conferred resistance to mobocertinib.

In conclusion, although our study was terminated early owing to futility, our results showed modest clinical activity of mobocertinib in Japanese patients with NSCLC harboring *EGFR* exon 20 insertion mutations and did not raise any additional safety concerns. Lastly, development of mobocertinib has now been terminated and market authorization has been voluntarily withdrawn (as of October 2023) from all relevant countries based on the results of the phase 3 EXCLAIM-2 study (NCT04129502).

## Supplementary Information

Below is the link to the electronic supplementary material.

Supplementary file1 (PDF 174 KB)

## Data Availability

The data sets, including the redacted study protocol, redacted statistical analysis plan, and individual participant data supporting the results reported in this article, will be made available within three months from initial request to researchers who provide a methodologically sound proposal. The data will be provided after de-identification, in compliance with applicable privacy laws, data protection, and requirements for consent and anonymization.
