# Supplementary material for: A phase 2 study of mobocertinib as first-line treatment in Japanese patients with non-small cell lung cancer harboring EGFR exon 20 insertion mutations
Source: Int J Clin Oncol. 2024 Aug 27;29(10):1461–74. doi: 10.1007/s10147-024-02588-y (PMC11420270; doi:10.1007/s10147-024-02588-y)
Supplement: Supplementary file 1 — Supplementary file1 (PDF 174 KB) [file 10147_2024_2588_MOESM1_ESM.pdf]

Supplemental Appendix

Title: A phase 2 study of mobocertinib as first-line treatment in Japanese patients with non-small cell lung cancer harboring *EGFR* exon 20 insertion mutations

Journal name: International Journal of Clinical Oncology

Author names: Kiyotaka Yoh ·Koichi Azuma ·Hidetoshi Hayashi ·Makoto Nishio ·Kenichi Chikamori ·Eiki Ichihara ·Yasutaka Watanabe ·Takayuki Asato ·Tadayuki Kitagawa ·Robert J. Fram ·Yuichiro Ohe

Corresponding author: Yuichiro Ohe MD PhD

Address: Department of Thoracic Oncology, National Cancer Center Hospital, 5-1-1 Tsukiji, Chuo-ku, Tokyo, 104-0045 Japan

Email: yohe@ncc.go.jp

Table S1 Overview of AEs[a] (safety population)

AE category, *n* (%)	**Mobocertinib 160 mg QD (*N* = 33)**
Any AE	33 (100.0)
Drug-related AEs	33 (100.0)
AEs leading to dose modification	27 (81.8)
Discontinuation	5 (15.2)
Reduction	20 (60.6)
Interruption	27 (81.8)
SAEs	11 (33.3)
Drug-related SAEs	6 (18.2)
AEs of grade 3 or higher	21 (63.6)
Drug-related AEs of grade 3 or higher	17 (51.5)
AEs resulting in death	2 (6.1)
Drug-related AEs resulting in death	0 (0.0)

[a]AEs that occurred after administration of the first dose of study drug and through 30 days after the last dose or before initiation of the subsequent anti-cancer therapies, whichever came first.

AE, adverse event; QD, once daily; SAE, serious adverse event.

Table S2 AEs[a] by SOC and PT (safety population)

AEs, *n* (%)	**Mobocertinib 160 mg QD (*N* = 33)**	
	**Any grade in ≥ 2 patients[b]**	**Grade 3 or higher**
Any AE	33 (100.0)	21 (63.6)
Blood and lymphatic system disorders	7 (21.2)	1 (3.0)
Anemia	6 (18.2)	–
Neutropenia	1 (3.0)	1 (3.0)
Cardiac disorders	2 (6.1)	1 (3.0)
Cardiac failure	2 (6.1)	1 (3.0)
Eye disorders	4 (12.1)	–
Dry eye	2 (6.1)	–
Gastrointestinal disorders	33 (100.0)	10 (30.3)
Diarrhea	33 (100.0)	7 (21.2)
Stomatitis	21 (63.6)	–
Nausea	19 (57.6)	1 (3.0)
Vomiting	7 (21.2)	–
Constipation	6 (18.2)	–
Dental caries	3 (9.1)	–
Dry mouth	2 (6.1)	–
Gastrointestinal mucosal disorder	1 (3.0)	1 (3.0)
Pancreatitis acute	1 (3.0)	1 (3.0)
General disorders and administration site conditions	12 (36.4)	–
Pyrexia	4 (12.1)	–
Chest pain	3 (9.1)	–
Malaise	3 (9.1)	–

AEs, *n* (%)	Mobocertinib 160 mg QD (*N* = 33)	
	Any grade in ≥ 2 patients[b]	Grade 3 or higher
Fatigue	2 (6.1)	–
Immune system disorders	2 (6.1)	1 (3.0)
Anaphylactic shock	1 (3.0)	1 (3.0)
Infections and infestations	23 (69.7)	1 (3.0)
Paronychia	21 (63.6)	–
Conjunctivitis	3 (9.1)	–
Pneumonia	3 (9.1)	1 (3.0)
Nasopharyngitis	2 (6.1)	–
Investigations	24 (72.7)	11 (33.3)
Lipase increased	13 (39.4)	3 (9.1)
Weight decreased	13 (39.4)	2 (6.1)
Amylase increased	9 (27.3)	1 (3.0)
Blood creatinine increased	8 (24.2)	–
Alanine aminotransferase increased	6 (18.2)	4 (12.1)
Electrocardiogram QT prolonged	6 (18.2)	2 (6.1)
Aspartate aminotransferase increased	5 (15.2)	1 (3.0)
Lymphocyte count decreased	5 (15.2)	3 (9.1)
Platelet count decreased	2 (6.1)	1 (3.0)
White blood cell count decreased	2 (6.1)	–
Metabolism and nutrition disorders	12 (36.4)	4 (12.1)
Decreased appetite	11 (33.3)	2 (6.1)
Hypokalemia	2 (6.1)	2 (6.1)
Hyponatremia	1 (3.0)	1 (3.0)

AEs, *n* (%)	**Mobocertinib 160 mg QD (*N* = 33)**	
	**Any grade in ≥ 2 patients[b]**	**Grade 3 or higher**
Musculoskeletal and connective tissue disorders	10 (30.3)	–
Arthralgia	2 (6.1)	–
Muscle spasms	2 (6.1)	–
Neoplasms benign, malignant and unspecified (including cysts and polyps)	1 (3.0)	1 (3.0)
NSCLC	1 (3.0)	1 (3.0)
Nervous system disorders	11 (33.3)	1 (3.0)
Dysgeusia	6 (18.2)	–
Cerebral infarction	1 (3.0)	1 (3.0)
Psychiatric disorders	4 (12.1)	1 (3.0)
Insomnia	3 (9.1)	–
Delirium	1 (3.0)	1 (3.0)
Respiratory, thoracic, and mediastinal disorders	11 (33.3)	1 (3.0)
Pneumonitis	3 (9.1)	–
Nasal inflammation	2 (6.1)	–
Oropharyngeal pain	2 (6.1)	–
Pulmonary embolism	1 (3.0)	1 (3.0)
Skin and subcutaneous tissue disorders	33 (100.0)	2 (6.1)
Dermatitis acneiform	14 (42.4)	1 (3.0)
Rash	14 (42.4)	–
Dry skin	13 (39.4)	1 (3.0)
Rash maculo-papular	7 (21.2)	–

AEs, **n** (%)	**Mobocertinib 160 mg QD (N = 33)**	
	**Any grade in ≥ 2 patients[b]**	**Grade 3 or higher**
Palmar-plantar erythrodysesthesia syndrome	3 (9.1)	–
Alopecia	2 (6.1)	–
Dermatitis	2 (6.1)	–
Dermatitis contact	2 (6.1)	–
Eczema asteatotic	2 (6.1)	–
Pruritus	2 (6.1)	–
Urticaria	2 (6.1)	–
Vascular disorders	7 (21.2)	–
Hypertension	4 (12.1)	–

[a]AEs that occurred after administration of the first dose of study drug and through 30 days after the last dose or before initiation of the subsequent anti-cancer therapies, whichever came first.

[b]AEs occurring in < 2 patients are also included if they are presented in the 'Grade 3 or higher' column.

AE, adverse event; NSCLC, non-small cell lung cancer; PT, preferred term; QD, once daily; QT, QT interval; SOC, system organ class.
